# Persisting lung pathogenesis and minimum residual virus in hamster after acute COVID-19

**DOI:** 10.1007/s13238-021-00874-3

**Published:** 2021-09-07

**Authors:** Lunzhi Yuan, Huachen Zhu, Ming Zhou, Jian Ma, Rirong Chen, Liuqin Yu, Wenjia Chen, Wenshan Hong, Jia Wang, Yao Chen, Kun Wu, Wangheng Hou, Yali Zhang, Shengxiang Ge, Yixin Chen, Quan Yuan, Qiyi Tang, Tong Cheng, Yi Guan, Ningshao Xia

**Affiliations:** 1 State Key Laboratory of Molecular Vaccinology and Molecular Diagnostics, National Institute of Diagnostics and Vaccine Development in Infectious Diseases, School of Life Sciences, School of Public Health, Xiamen University, Xiamen, 361000 China; 2 State Key Laboratory of Emerging Infectious Diseases, The University of Hong Kong, Hong Kong, China; 3 Joint Institute of Virology (Shantou University and The University of Hong Kong), Guangdong-Hongkong Joint Laboratory of Emerging Infectious Diseases, Shantou University, Shantou, 515063 China; 4 Department of Microbiology, Howard University College of Medicine, Washington, DC 20059 USA; 5 Research Unit of Frontier Technology of Structural Vaccinology, Chinese Academy of Medical Sciences, Xiamen, 361102 China

**Dear Editor,**

Severe acute respiratory syndrome coronavirus 2 (SARS-CoV-2) has infected more than 200 million people, causing coronavirus disease 2019 (COVID-19) worldwide. Lungs are the primary target organ of SARS-CoV-2 infection. The mild COVID-19 cases develop symptoms of fever, fatigue, muscle weakness, chest pain, headache and cough (Chen et al., 2020; Wang et al., 2020; Zhu et al., 2020), while severe COIVD-19 cases might have pneumonia, breathing difficulties, multiple organ failure and death (Chen et al., 2020; Wang et al., 2020; Zhu et al., 2020). Both the clinicians and researchers have largely focused on the acute phase of COVID-19, but the long-term health consequences of the COVID-19 patients after clinical recovery remain less investigated. Several clinical cohorts demonstrated that some patients discharged from hospital still experience symptoms including fatigue, muscle weakness, chest pain, cough and breathing difficulties (Carfi et al., 2020; Lim et al., 2020; Huang et al., 2021). Moreover, severely impaired pulmonary diffusion capacities and abnormal chest imaging manifestations were observed in some convalescent patients (Huang et al., 2021). Surprisingly, residual virus (An et al., 2020; Yao et al., 2020; Kim et al., 2021) were detected in some convalescent patients.

The above mentioned clinical observations led to our further investigation into the persisting lung pathogenesis and residual virus in the host after the acute COVID-19. The Syrian hamster has been successfully used as model for SARS-CoV-2 infection (Chan et al., 2020; Imai et al., 2020; Sia et al., 2020; Yuan et al., 2021). In order to know whether the infection-caused body weight loss, lung pathogenesis and the production of neutralizing antibody is viral dose-dependent, male hamsters were intranasally infected with 1 × 10^2^, 1 × 10^3^, 1 × 10^4^ or 1 × 10^5^ PFU of SARS-CoV-2, respectively (Fig. S1A). All of the hamsters showed significant body weight loss from 5 to 7 days post infection (dpi) (Fig. S1B). Infectious SARS-CoV-2 was detected from lung lobes at 5 dpi by using a standard cytopathic effect (CPE) based titration method in 96-well plate, and undetectable at 7 dpi (Fig. S1C). Serum neutralizing antibody was detected at 7 dpi (Fig. S1D). Immunohistochemistry staining results showed diffusive distribution of SARS-CoV-2 nucleocapsid protein (NP) in positively infected cells in lung lobes of these hamsters at 5 dpi (Fig. S1E). Taken together, both low and high doses of SARS-CoV-2 challenge can establish a productive infection and induce rapid humoral immune response in hamsters.

To know the animal health statues after being recovered from acute COVID-19, we performed experiments as shown in Fig. 1. We used male hamster in this experiment because male hamsters are more susceptible to SARS-CoV-2 than female ones. Male hamsters (3/group) were intranasally infected with SARS-CoV-2 at 4 different doses and observed for 42 days (Fig. 1A). Most of the hamsters showed a recovery of body weight from 7 dpi, while some of them showed persisting body weight loss as observed till 42 dpi (Fig. 1B). Hamsters without SARS-CoV-2 infection showed an increasing body weight from 0 to 42 dpi (Fig. S2A). Remarkably, a rebound of viral RNA in nasal washings were detected by quantitative real-time polymerase chain reaction (qRT-PCR) during the convalescent phase (Fig. S2B). Infectious SARS-CoV-2 was not detected from lung tissues at 42 dpi by a standard CPE based titration method in 96-well plates (Fig. 1C). Then, homogenized lung tissues were seeded on a 24-well plate, cultured with Vero-E6 cells for 7 days and the cells were harvested for titration of SARS-CoV-2 (Fig. 1D). We found that 6 out of 12 hamsters were positive in containing infectious viral particles in in their lungs at 7 dpi (Fig. 1E). We also examined the serum neutralization antibodies and found that serum neutralizing antibody titers of the infected hamsters increased from 7 to 21 dpi and decreased from 21 dpi (Fig. 1F), which indicates an early decline of humoral immune response against SARS-CoV-2. Immunohistochemistry staining results demonstrated scattered distribution of SARS-CoV-2 NP positive cells in lung lobes of the hamsters (Figs. 1G and S2C). These results provided evidence that a minimum number of SARS-CoV-2 particles exist in the lung lobes of hamsters after being recovered from acute COVID-19, which is consistent to the clinical findings that residual viruses were detected in lung tissues of a convalescent patient (Yao et al., 2020). Taken together, persisting body weight loss was observed in hamster BC54, BC56, BC59, BC61, and BC63 (Fig. 1B), intermittent viral shedding was detected in the nasal washings of hamster BC53, BC54, BC56, BC57, BC59, BC60, BC61 and BC63 (Fig. S2B), and viral particles were detected in liver tissues of hamster BC54, BC56, BC59, BC60, BC61 and BC62 (Fig. S2C). Therefore, these data suggested that all five hamsters (BC54, BC56, BC59, BC61, and BC63) with persisting body weight loss showed detectable intermittent viral shedding and four showed residual virus in lung tissues. Notably, the hamsters with detectable SARS-CoV-2 in lung tissues showed a lower serum neutralizing antibody titers than the negative ones (Fig. S2D), suggesting that high serum neutralizing antibody titer is essential for prevention of viral rebound after acute phase of COVID-19.

**Figure 1:**
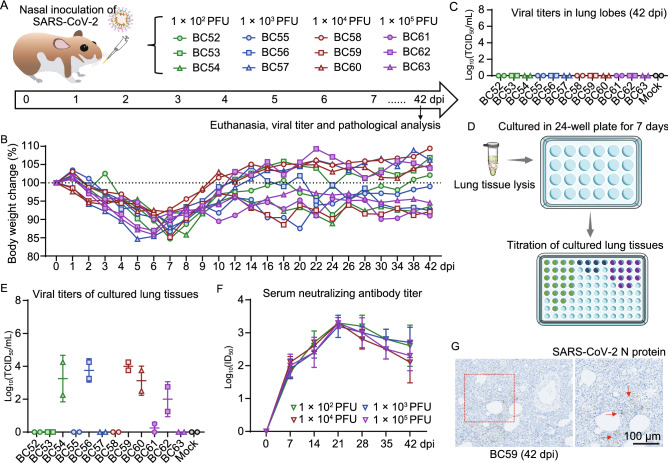
**Acute infection and recovery of hamsters with SARS-CoV-2**. (A) Schematic diagram of SARS-CoV-2 infection and animal operations for observation of persisting symptoms after acute SARS-CoV-2 infection. Hamsters were intranasally inoculated with 1 × 10^2^ to 1 × 10^5^ PFU of SARS-CoV-2, respectively (*n* = 3/group). All of the animals were euthanized at 42 dpi for virological and histological analysis. (B) Body weight changes after SARS-CoV-2 infection from 0 to 7 dpi (*n* = 3/group). (C) Titers of live virus in lung tissues collected at 42 dpi by a CPE-based titration assay in 96-well plates. For each hamster, one near-hilum lung tissue sample and one away-hilum lung tissue sample were used for titration. (D) The samples collected at 42 dpi were incubated with Vero-E6 cells in 24-well plates for a 7-day culture. (E) And then, the Vero-E6 cells were collected for a second-round titration. (F) Titers of serum neutralizing antibody levels from 0 to 42 dpi (*n* = 3/group). There is no significant difference between the NAb titers of different groups at 42 dpi (1 × 10^2^ PFU vs. 1 × 10^3^ PFU, *P* > 0.05; 1 × 10^2^ PFU vs. 1 × 10^4^ PFU, *P* > 0.05; 1 × 10^2^ PFU vs. 1 × 10^5^ PFU, *P* > 0.05). (G) Representative image of immunohistochemistry staining for SARS-CoV-2 NP in lung lobe sections collected at 42 dpi (left). The area in the scattered square was enlarged (bar = 100 μm), the arrows points to the NP positive cells

Next, the lung lobes of SARS-CoV-2 infected hamsters at 5, 7 and 42 dpi were collected for a systematically pathological analysis. The results of hematoxylin & eosin (HE) staining for lung lobes of hamsters at 5 and 7 dpi showed typical features of severe pneumonia including increasing lung lobe consolidation and alveolar destruction, diffuse inflammation, protein-rich fluid exudate, hyaline membrane formation and severe pulmonary haemorrhage (Figs. 2A, 2B, S3A, and S3B). The H&E staining of lung lobes collected at 42 dpi (BC52, BC53, BC57, BC58, BC60 and BC62) showed a pathology recovery from the severe pneumonia and lung injury (Figs. 2C and S3C). However, varied degrees of lung lesions were detected in one or several lobes (marked by yellow asterisk) of hamsters include BC54, BC55, BC56, BC59, BC61 and BC63 (Figs. 2C and S3C). Mock hamster without infection showed a normal lung lobe structure (Fig. S3D). The severity of lung pathogenesis is quantified by comprehensive pathological score based on alveolar septum thickening and consolidation, hemorrhage, exudation, pulmonary edema and mucous, recruitment and infiltration of inflammatory immune cells in hamster lung lobes. Unfortunately, both low and high doses of SARS-CoV-2 challenge can cause a persisting lung injury through 42 dpi. Although the hamster lung lobes collected at 42 dpi showed lower average pathological score than the ones collected at 5 and 7 dpi, several lung lobes of the convalescent hamsters still had a high pathological score, suggesting they cannot fully recover from the severe lung injury induced by SARS-CoV-2 infection within 42 dpi. Interestingly, the end-point body weight changes of infected hamsters have a linear relationship between their lung pathological score at 5, 7 and 42 dpi (Fig. 2F). These data suggest that body weight loss is still an important indicator of lung injury after acute COVID-19. In the hamster model, the recovery of body weight after acute SARS-CoV-2 infection indicate a restoration of severe lung injury.

**Figure 2:**
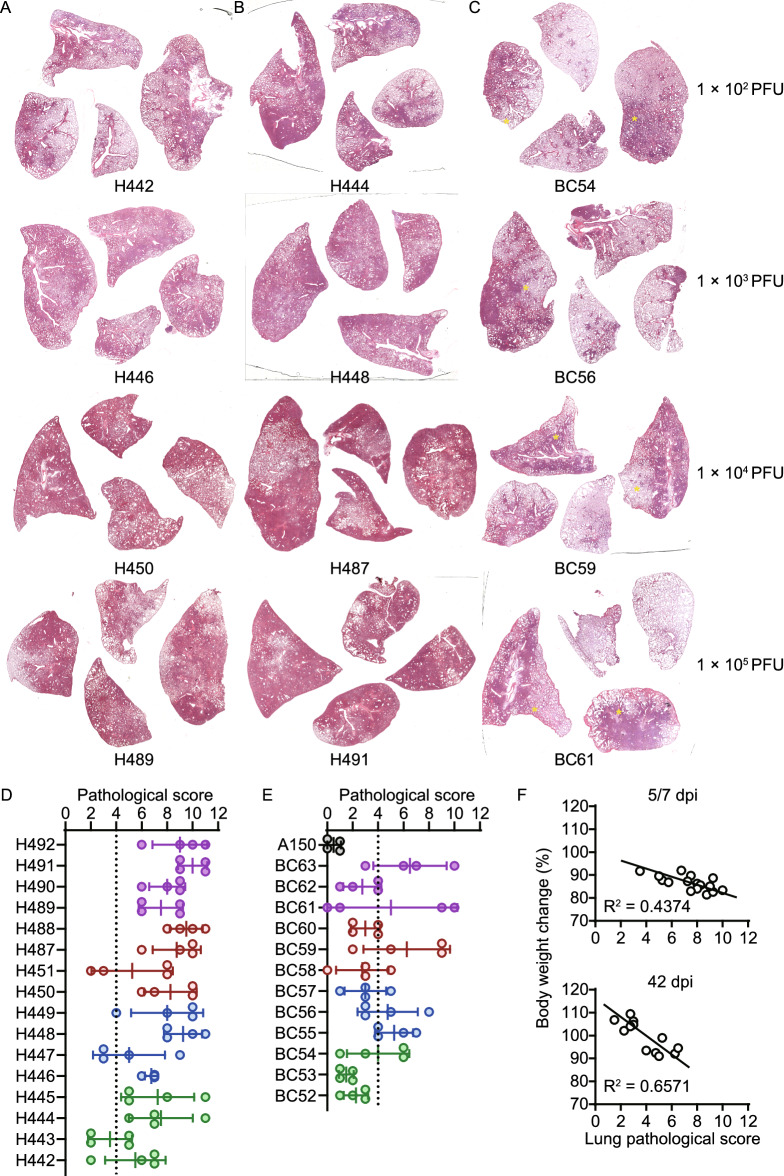
**Pathological analysis of lung lobe tissues collected from hamsters in acute SARS-CoV-2 infection phase and convalescent phase**. For each hamster, four lung lobes were fixed in formalin for pathological analysis. Representative H&E staining for lung lobe sections collected from SARS-CoV-2 infected hamsters at (A) 5, (B) 7 and (C) 42 dpi, respectively. These hamsters were infected with 1 × 10^2^ PFU (line 1), 1 × 10^3^ PFU (line 2), 1 × 10^2^ PFU (line 3) and 1 × 10^5^ PFU (line 4) of SARS-CoV-2, respectively. H&E staining for all the rest hamsters were shown in Fig. S3. Comprehensive pathological scores for lung of hamsters during (D) the acute SARS-CoV-2 infection phase and (E) the convalescent phase. Scores were determined based on the severity and percentage of injured areas for each lung lobe. There are significant differences between the lung pathological scores of different groups at 5 dpi (1 × 10^2^ PFU vs. 1 × 10^3^ PFU, *P* = 0.048; 1 × 10^2^ PFU vs. 1 × 10^4^ PFU, *P* > 0.05; 1 × 10^2^ PFU vs. 1 × 10^5^ PFU, *P* = 0.022). There is no significant difference between the lung pathological scores of different groups at 7 and 42 dpi (1 × 10^2^ PFU vs. 1 × 10^3^ PFU, *P* > 0.05; 1 × 10^2^ PFU vs. 1 × 10^4^ PFU, *P* > 0.05; 1 × 10^2^ PFU vs. 1 × 10^5^ PFU, *P* > 0.05). (F) The linear relationship between body weight change and average lung pathological score of each individual hamster

As the pandemic of SARS-CoV-2 is still ongoing, the viral rebound and persisting symptoms among the clinically recovered patients might become a challenge to the public health. The underlying mechanism of persisting symptoms, “long term positive” and “recurrent positive” cases of COVID-19 is unclear and might be multifactorial. A recent cohort study revealed that the patients with a more severe COVID-19 have increased risk of pulmonary diffusion abnormality, fatigue or muscle weakness, and anxiety or depression in the six months after discharge (Huang et al., 2021). Among 349 discharged patients, the proportion of participants with lung diffusion impairment was 22% (18 of 83) for scale 3, 29% (48 of 165) for scale 4, and 56% (48 of 86) for scale 5–6 of pulmonary diffusion abnormality six months after symptom onset (Huang et al., 2021). Moreover, seropositivity and titers of the neutralizing antibodies in these were significantly lower than at acute phase (Huang et al., 2021). Decrease of neutralizing antibody titer is also found in the hamsters after acute SARS-CoV-2 infection. Meanwhile, the reports of “long-positive” and “recurrent positive” COVID-19 cases in discharged human patients (An et al., 2020; Yao et al., 2020; Kim et al., 2021), and the minimum residual virus in lung of hamsters indicate the risk of SARS-CoV-2 re-infection among the populations with low neutralizing antibody titer.

Delineating the natural history of COVID-19 in experimental animal model is important to improve our clinical management of the patients (Yuan et al., 2020). The hamsters with intermittent viral shedding in nasal washings are similar to the recurrent positive patients. We speculate the residual virus in the lung tissue might be the cause of intermittent viral shedding in nasal washings. Furthermore, the residual virus is likely to cause lung pathogenesis when it replicates. Serum neutralizing antibody plays a critical role in preventing the replication of the residual SARS-CoV-2. However, hamsters showed a gradually decreasing neutralizing antibody levels in serum from 21 to 42 dpi and a minimum residual virus was detectable in lung tissue of the hamsters with lower serum neutralizing antibody at 42 dpi. Altogether, the dynamics and relationships between body weight loss, antibody titers, residual virus and lung pathological scores of hamsters revealed the complicated virological and pathological changes during the convalescent phase of SARS-COV-2 infection, and implying the convalescent patients with low serum neutralizing antibody levels might be inadequate to resist viral rebound and re-challenge of SARS-CoV-2. Therefore, a long-term serological investigation, monitoring of pulmonary functions and vaccination are necessary for the convalescent patients.

## Supplementary Information

Below is the link to the electronic supplementary material.

Supplementary file1 (PDF 1067 kb)
