# Supplementary material for: Persisting lung pathogenesis and minimum residual virus in hamster after acute COVID-19
Source: Protein Cell. 2021 Sep 7;13(1):72–7. doi: 10.1007/s13238-021-00874-3 (PMC8422370; doi:10.1007/s13238-021-00874-3)
Supplement: Supplementary file 1 — Supplementary file1 (PDF 1067 kb) [file 13238_2021_874_MOESM1_ESM.pdf]

Supplementary information

Persisting lung pathogenesis and minimum residual virus in hamster after acute COVID-19

Lunzhi Yuan, Huachen Zhu, Ming Zhou, Jian Ma, Rirong Chen, Liuqin Yu, Wenjia Chen, Wenshan Hong, Jia Wang, Yao Chen, Kun Wu, Wangheng Hou, Yali Zhang, Shengxiang Ge, Yixin Chen, Quan Yuan, Qiyi Tang, Tong Cheng, Yi Guan and Ningshao Xia

KEY RESOURCES TABLE

REAGENT or RESOURCE	SOURCE	IDENTIFIER
Antibodies		
Mouse Anti-SARS-CoV-2 NP	House keeping	15A7-1
Virus and cells		
SARS-CoV-2	This study	AP-8
Vero-E6 cells	ATCC	#CCL-81
Biological Samples		
Hamster nasal washings<br>Hamster serum samples<br>Homogenized lung tissues<br>Hamster lung tissue paraffin sections	This study	N/A
Reagents		
Dulbecco's Modified Eagle Medium (DMEM)	GIBCO	#11995
Fetal bovine serum (FBS)	GIBCO	#10270106
Phosphate buffer saline (PBS)	GIBCO	#10010031
TPCK-trypsin	SIGMA-ALDRICH	#T1426
Penicillin-Streptomycin	Invitrogen	#15140-122
MgCl2	Thermo Fisher	#AM9530G
Isoflurane	RWD Life Science	#R510-22
QIAamp Viral RNA Mini kit	Qiagen	#52906
COVID-19 qRT-PCR Kit	Wantai, Beijing, China	N/A
Immunohistochemistry kit		#KIT-9730

Hematoxylin<br>Eosin	Maxim Biotechnology,<br>Fuzhou, China	#CTS-1096<br>#CTS-4094

EXPERIMENTAL MODEL AND SUBJECT DETAILS

Experimental Animals. The Golden Syrian Hamster was derived from Charles River Laboratories and raised at the specific pathogen free animal feeding facilities. All the animal experiments were approved by the Medical Ethics Committee (SUCM2021-112). All experiments with infectious SARS-CoV-2 were performed in the biosafety level 3 (BSL-3) and animal biosafety level 3 (ABSL-3) facilities affiliated to the State Key Laboratory of Emerging Infectious Diseases, School of Public Health, The University of Hong Kong. Our staff wear powered air-purifying respirators that filter the air, and disposable coveralls when they culture the virus and handle animals that are in isolators. The researchers are disinfected before they leave the room and then shower on exiting the facility. All facilities, procedures, training records, safety drills, and inventory records are subject to periodic inspections and ongoing oversight by the institutional biosafety officers who consult frequently with the facility managers.

Virus Stocks. The SARS-CoV-2 virus (hCoV-19/China/AP8/2020) was isolated from a 32 year-old male patient returning from Wuhan in late January 2020, and passaged on Vero-E6 cells. The AP8 strain (GISAID Accession number EPI_ISL_1655937). Viral stocks were prepared in Vero-E6 cells with DMEM containing 2% FBS, 5ug/mL TPCK-trypsin, Penicillin-Streptomycin and 30mmol/L $MgCl_2$. Viruses were harvested and the titers were determined by means of plaque assay in Vero-E6 cells. Viral stocks (fourth-passage, 1.36×10^6 PFU/mL, 1 mL per stock) were stored in ultra-low temperature refrigerator.

Virus Inoculation and Sample Collection. The hamsters were anesthetized by isoflurane and the nasally inoculated with 1×10^4 PFU dose of SARS-CoV-2 diluted in

200uL PBS. Body weight of these hamsters were measured by electronic balance. Nasal washings were collected to exam virus shedding via respiratory tract. Hamsters were treated with isoflurane lightly, after that, 500μL of PBS was injected into one nasal opening while collecting the turbid wash from the other one without any blood contamination. Blood was collected for detection of serum neutralizing antibody. Hamsters were treated with isoflurane lightly, after that, capillary tube was used to collect blood from orbital vein. During long-term observation of the hamsters, the IVC cages, bedding material, food, water and operation tools were renewed weekly to avoid pollution of viral RNA. Hamsters were euthanized at indicated time point for detection of viral load and analysis of pathogenesis in lung lobes.

Titration Assay and qRT-PCR to Detect Viral Load. To quantitate the viral RNA copies in the nasal washings and homogenized lung tissues, viral RNA was extracted by using a QIAamp Viral RNA Mini kit (Qiagen, Hilden, Germany) according to the manufacturer's instructions. The qRT-PCR was conducted by using the SLAN-96S Real-Time System (Hongshi, Shanghai, China) with a COVID-19 qRT-PCR Kit from Wantai (Beijing, China). Relative Viral RNA of SARS-CoV-2 N protein was determined using primers pairs and probes shown in the kit instruction. Viral RNA copies were expressed on a $\log_{10}$ scale after normalized to the standard curve obtained by using ten-fold dilutions of a SARS-CoV-2 stock with known viral titer. Titer of live virus in homogenized lung tissues and cell cultures were measured by standard $TCID_{50}$ method in Vero-E6 cells seeded in 96-well plate. In brief, the samples were gradient diluted, added into the 96-well plate and incubate with the Vero-E6 cells for one hour. Three days after incubation, the cytopathic effects were observed and used for calculation of the viral titers.

Measurement of Serum Neutralizing Antibody. Hamster serum samples were

gradient diluted and incubated with 100 $TCID_{50}$ of SARS-CoV-2 for one hour. And then, the mix was added into 96-well plate seed with Vero-E6 cells for another one hour incubation. After that, Three days after incubation, the inhibition of cytopathic effects were observed and used for calculation of the serum neutralizing antibody titer.

Histopathological Studies. For pathological analysis, lung tissues were fixed in formalin for 48 hours, dehydrated and then embedded in paraffin wax. The wax block of lung tissues were into 4μm sections for several pathological staining and analysis. H&E staining was employed for analysis of general lung pathogenic lesions include pulmonary edema, consolidation and inflammation. The standards for pathological score of lung tissues in this study are derived from our previous study in hamster model. Comprehensive pathological score of lung sections were performed according to the degree of lung lesions include alveolar septum hyperplasia, consolidation and impairment of alveolar structure, fluid exudation, mucus suppository, thrombus, inflammation recruitment and infiltration of immune cells in each individual lung lobes. For each hamster, three or four lung lobes were employed for evaluation of comprehensive pathological score. In brief, H&E staining result of each lung lobe was analyzed for its severity of pathological change. The pathological score include: a) Alveolar septum thickening and consolidation; b) Hemorrhage, exudation, pulmonary edema and mucous; c) Recruitment and infiltration of inflammatory immune cells. For each issue, score related to the severity: 0 indicate no pathological change was observed, 1 indicate moderate pathological change, 2 indicate mild pathological change, 3 indicate severe pathological change and 4 indicate very severe pathological change. In conclusion, scores of such three issues were added as the comprehensive pathological score of a lung lobe, and the average comprehensive pathological score of the lobes indicate the severity of lung pathogenesis in an evaluated hamster.

Immunohistochemistry staining for SARS-CoV-2 nucleocapsid protein (NP) was employed for analysis of viral antigen expression and distribution in lung tissues. A mouse anti SARS-CoV-2 N protein specific antibody (15A7-1, house-keeping) was used as the first antibody of immunohistochemistry staining. The pathological reagents include immunohistochemistry kit (#KIT-9730), Hematoxylin (#CTS-1096) and Eosin (#CTS-4094) were purchased from Maxim Biotechnology (Fuzhou, China). The images of whole lung lobes were screened by a high-throughput screening microscope system (EVOS M7000, Invitrogen of Thermo Fisher Scientific). The high magnification images were taken by a high-resolution microscope (AXIO Imagier.A2, Zeiss).

Statistical Analysis. Student's unpaired two-tailed t-test and one-way ANOVA were performed using GraphPad Prism 8.0 (GraphPad Software). Data are presented as the means ± SD. Two-sided p-values <0.05 were considered significant: *P <0.05, **P <0.01, ***P <0.001, *NS* indicates no significance.

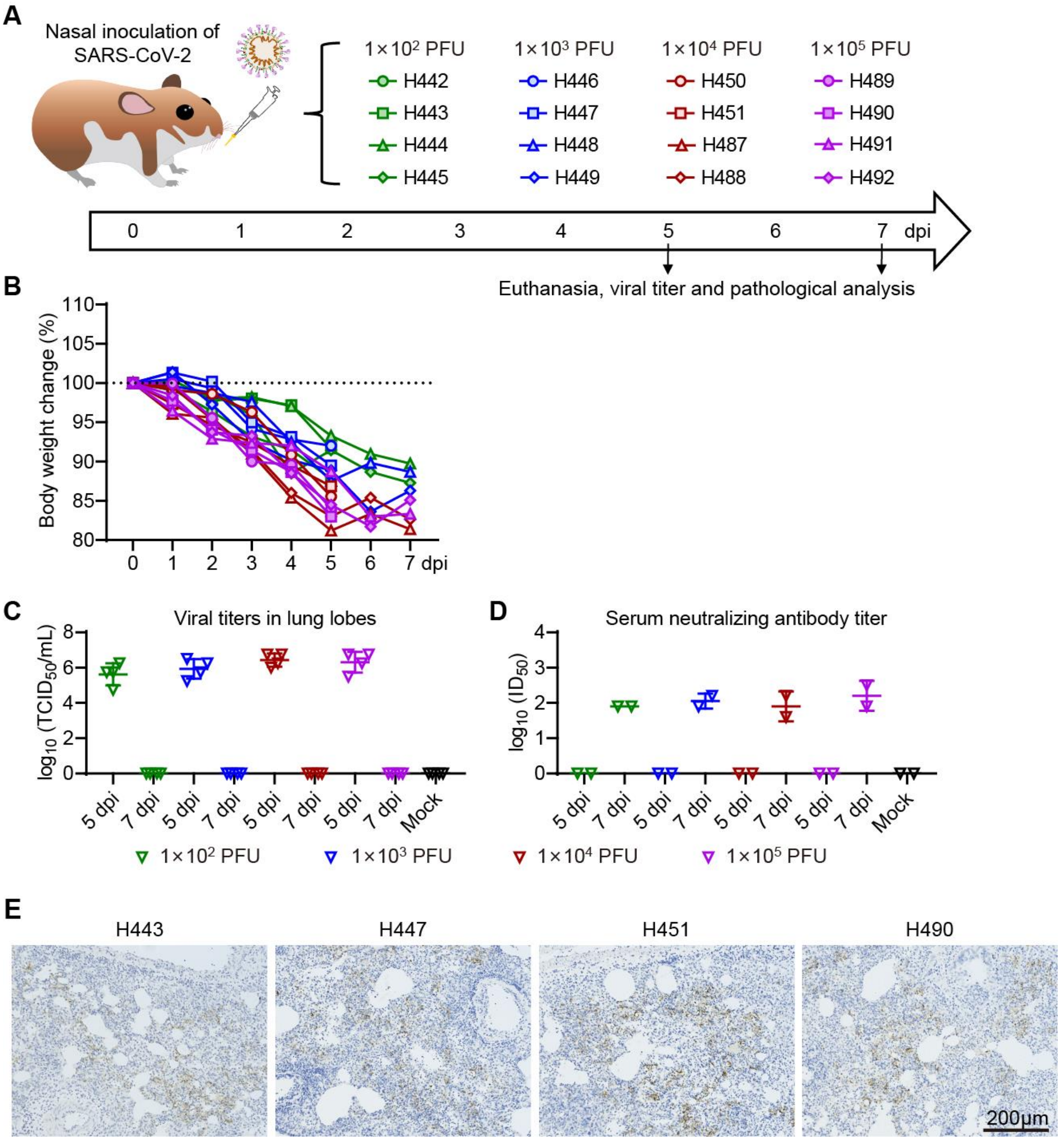

Figure S1. Acute SARS-CoV-2 infection in hamsters. (A) Schematic diagram of SARS-CoV-2 infection and animal operations for acute SARS-CoV-2 infection. Hamsters were intranasally inoculated with 1×10^2 to 1×10^5 PFU of SARS-CoV-2, respectively (n=4/group). In brief, H442, H443, H444, H445 in 1×10^2 PFU group, H446, H447, H448, H449 in 1×10^5 PFU group, H450, H451, H487, H488 in 1×10^4 PFU group, H489, H490, H491, H492 in 1×10^5 PFU group. Body weight were daily observed. Animals were euthanized at 5 and 7 dpi for virological and histological analysis. **(B)** Body weight changes of SARS-CoV-2 from 0 to 7 dpi (n=4/group). **(C)** Titers of live

virus in homogenized lung tissues collected at 5 and 7 dpi by a CPE-based titration assay in 96-well plates (n=4/group). For each hamster, one near-hilum lung tissue sample and one away-hilum lung tissue sample were used for titration. There is no significant different between the viral titers of different groups at 5 dpi (1×10^2 PFU vs. 1×10^3 PFU, $p>0.05$; 1×10^2 PFU vs. 1×10^4 PFU, $p>0.05$; 1×10^2 PFU vs. 1×10^5 PFU, $p>0.05$) **(D)** Titers of serum neutralizing antibody at 5 and 7 dpi (n=2/group). There is no significant different between the NAb titers of different groups at 5 dpi (1×10^2 PFU vs. 1×10^3 PFU, $p>0.05$; 1×10^2 PFU vs. 1×10^4 PFU, $p>0.05$; 1×10^2 PFU vs. 1×10^5 PFU, $p>0.05$). **(E)** Immunohistochemistry staining for SARS-CoV-2 NP positive cells in lung lobe sections of SARS-CoV-2 infected hamsters sacrificed at 5 dpi.

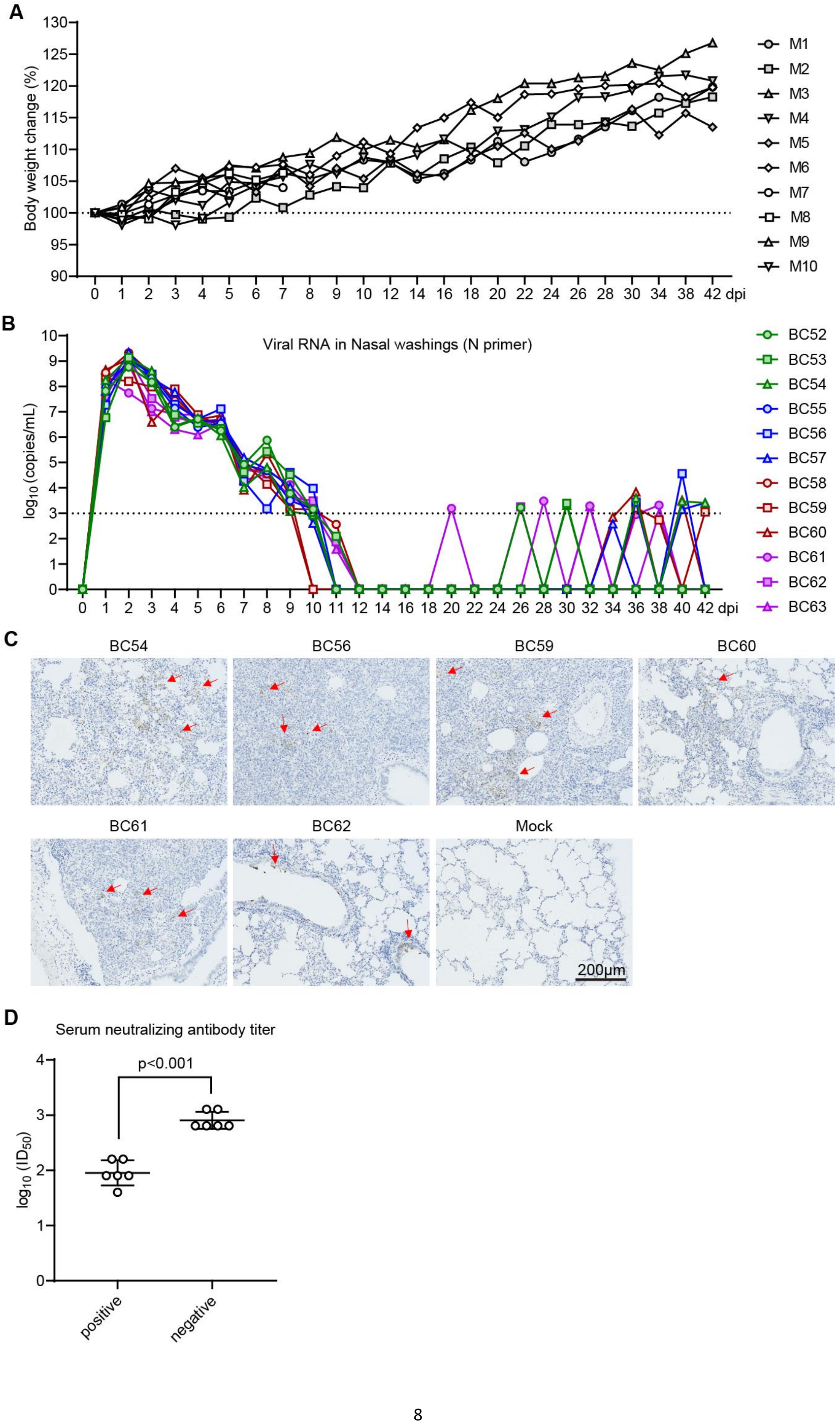
A
Body weight change (%)
130
125
120
115
110
105
100
95
90
0 1 2 3 4 5 6 7 8 9 10 12 14 16 18 20 22 24 26 28 30 34 38 42 dpi
M1
M2
M3
M4
M5
M6
M7
M8
M9
M10
B
Viral RNA in Nasal washings (N primer)
log10 (copies/mL)
10
9
8
7
6
5
4
3
2
1
0
0 1 2 3 4 5 6 7 8 9 10 11 12 14 16 18 20 22 24 26 28 30 32 34 36 38 40 42 dpi
BC52
BC53
BC54
BC55
BC56
BC57
BC58
BC59
BC60
BC61
BC62
BC63
C
BC54
BC56
BC59
BC60
BC61
BC62
Mock
200μm
D
Serum neutralizing antibody titer
p<0.001
log10 (ID50)
4
3
2
1
0
positive
negative

Figure S2. Extend information for virological features of persisting symptoms in hamsters after SARS-CoV-2 infection. (A) Body weight changes of mock hamsters without SARS-CoV-2 infection from 0 to 42 dpi (n=10/group). **(B)** Measurement of viral RNA levels in nasal washings collected from SARS-CoV-2 infected hamsters from 0 to 42 dpi by a qRT-PCR assay. Intermittent viral shedding were detectable in the nasal washings of hamster BC53, BC54, BC56, BC57, BC59, BC60, BC61 and BC63. **(C)** Immunohistochemistry staining for SARS-CoV-2 NP positive cells in lung lobe sections of SARS-CoV-2 infected hamsters sacrificed at 42 dpi. The images of BC59 was shown in Figure 1G as representative images, respectively. The lung lobe sections of mock hamster without SARS-CoV-2 infection was set as control (bar=200μm). **(D)** Detection for serum neutralizing antibody titers of SARS-CoV-2 infected hamsters sacrificed at 42 dpi. The hamsters with minimum residual virus (positive) in homogenized lung tissues (n=6) showed a lower level of average serum neutralizing antibody titer than the negative ones (n=6).

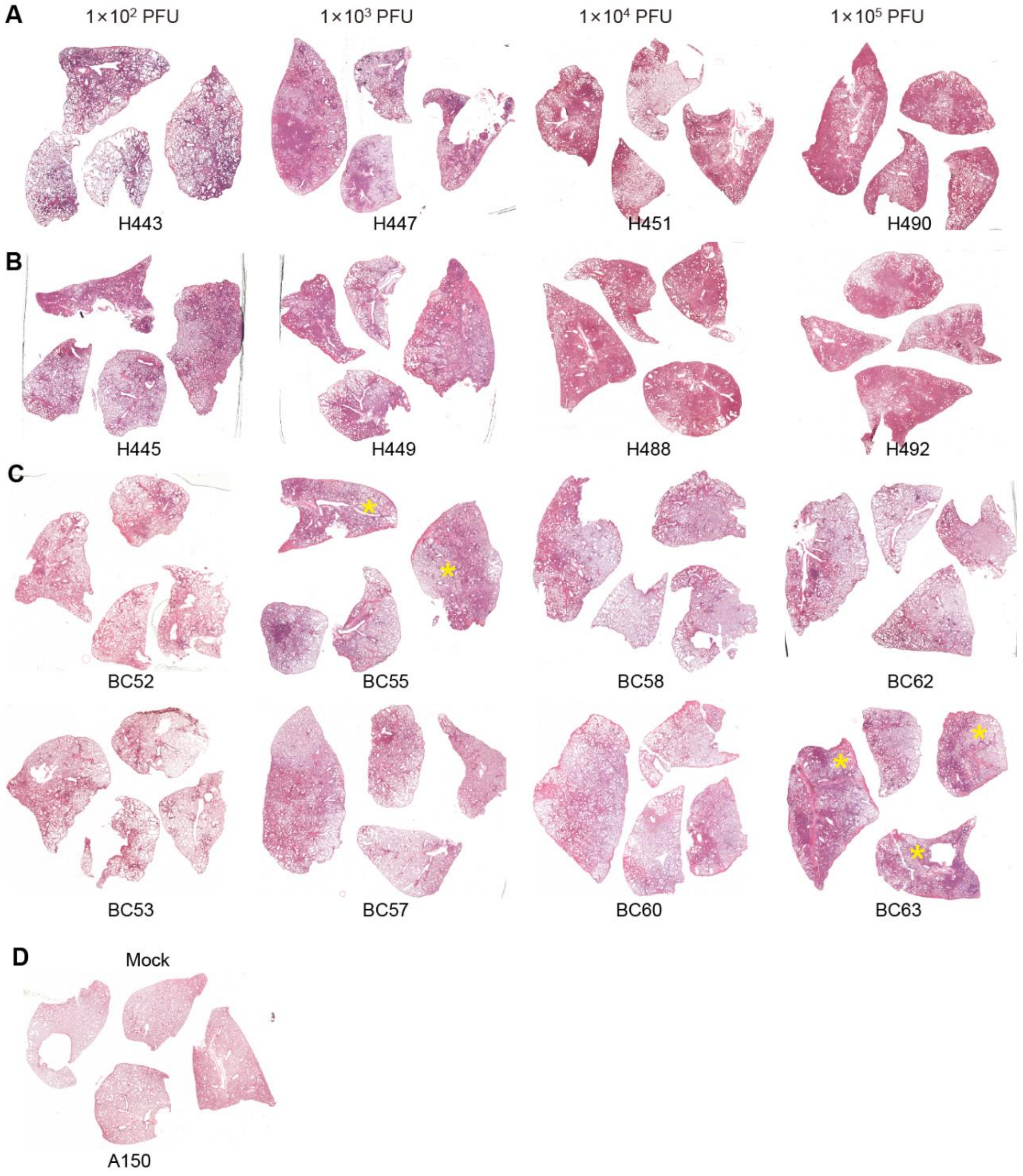

Figure S3. H&E staining of lung lobe tissues collected from hamsters in acute SARS-CoV-2 infection phase and convalescent phase. For each hamster, four lung lobes were fixed in formalin for pathological analysis. Representative H&E staining for lung lobe sections collected from SARS-CoV-2 infected hamsters at **(A)** 5, **(B)** 7 and **(C)** 42 dpi, respectively. These hamsters were infected with 1×10^2 PFU (line 1), 1×10^3 PFU (line 2), 1×10^2 PFU (line 3) and 1×10^5 PFU (line 4) of SARS-CoV-2, respectively.

(D) Lung lobe section of mock hamster without SARS-CoV-2 infection was set as control.

Table S1. Comprehensive pathological score of hamsters infected with SARS-CoV-2 sacrificed at different time points.

Dose of virus inoculation	Sacrifice time point	Label	Pathological lesions			Comprehensive pathological score of each individual lung lobe	Average pathological score
			Alveolar septum hyperplasia and consolidation	Pulmonary edema, hemorrhage and mucus suppository	Recruitment and infiltration of inflammatory cells		
10E2 PFU	42 dpi	BC52	1+1+1+0	1+1+1+1	1+1+0+0	3+3+2+1	2.25
		BC53	1+1+0+0	1+1+1+1	0+0+0+0	2+2+1+1	1.5
		BC54	2+2+1+0	2+2+1+1	2+2+1+0	6+6+3+1	4
10E3 PFU		BC55	2+2+1+1	2+2+2+2	3+2+1+1	7+6+4+4	5.25
		BC56	3+1+1+1	2+2+1+1	3+2+1+1	8+5+3+3	4.75
		BC57	1+1+1+0	2+1+2+1	2+1+0+0	5+3+3+1	3
10E4 PFU		BC58	2+1+1+0	2+1+1+0	1+1+1+0	5+3+3+0	2.75
		BC59	3+3+1+0	3+3+2+1	3+3+2+1	9+9+5+2	6.25
		BC60	1+1+0+0	1+1+1+1	2+2+1+1	4+4+2+2	3
10E5 PFU		BC61	3+3+0+0	3+3+1+0	3+4+0+0	9+10+1+0	5
		BC62	1+1+1+0	2+2+1+1	1+1+0+0	4+4+2+1	2.75
		BC63	3+3+2+1	4+2+2+1	3+2+2+1	10+7+6+3	6.5
10E2 PFU	5 dpi	H442	2+2+1+1	2+2+2+1	3+2+2+0	7+6+6+2	5.25
		H443	1+1+0+0	2+2+1+1	2+2+1+1	5+5+2+2	3.5

	7 dpi	H444	3+2+2+1	4+2+2+2	4+3+3+2	11+7+7+5	7.5
		H445	4+2+1+1	3+3+2+2	4+3+2+2	11+8+5+5	7.25
10E3 PFU	5 dpi	H446	2+2+2+1	2+2+2+2	3+3+3+3	7+7+7+6	6.75
		H447	3+2+1+1	3+1+1+1	3+2+1+1	9+5+3+3	5
	7 dpi	H448	3+3+2+2	4+3+3+3	4+4+3+3	11+10+8+8	9.25
		H449	3+3+2+1	3+3+1+1	4+4+3+2	10+10+8+4	8
10E4 PFU	5 dpi	H450	4+4+2+2	3+3+2+2	3+3+3+2	10+10+7+6	8.25
		H451	3+3+1+1	2+2+1+1	3+3+1+0	8+8+3+2	5.75
	7 dpi	H487	3+3+3+2	3+3+3+2	4+4+3+2	10+10+9+6	8.75
		H488	3+3+3+3	4+3+3+2	4+4+3+3	11+10+9+8	9.25
10E5 PFU	5 dpi	H489	3+3+2+2	3+3+3+2	3+3+3+2	9+9+8+6	8
		H490	3+3+2+2	3+3+2+2	3+3+2+2	9+9+6+6	7.5
	7 dpi	H491	3+3+3+3	4+4+3+3	4+4+3+3	11+11+9+9	10
		H492	3+3+3+2	4+3+3+2	4+4+3+2	11+10+9+6	9
Mock		A150	0+0+0+0	1+1+0+0	0+0+0+0	1+1+0+0	0.5
